# - LAA Occluder View for post-implantation Evaluation (LOVE) - standardized imaging proposal evaluating implanted left atrial appendage occlusion devices by cardiac computed tomography

**DOI:** 10.1186/s12880-016-0127-y

**Published:** 2016-03-24

**Authors:** Michael Behnes, Ibrahim Akin, Benjamin Sartorius, Christian Fastner, Ibrahim El-Battrawy, Martin Borggrefe, Holger Haubenreisser, Mathias Meyer, Stefan O. Schoenberg, Thomas Henzler

**Affiliations:** First Department of Medicine, University Medical Center Mannheim, Faculty of Medicine Mannheim, University of Heidelberg, Theodor-Kutzer-Ufer 1-3, 68167 Mannheim, Germany; Institute of Clinical Radiology and Nuclear Medicine, University Medical Center Mannheim, Faculty of Medicine Mannheim, University of Heidelberg, Theodor-Kutzer-Ufer 1-3, 68167 Mannheim, Germany

**Keywords:** LAA occlusion device, LAA occluder, Cardiac CTA, Peri-device leak, Lobe coverage, Neo-endothelialization, Thrombus

## Abstract

**Background:**

A standardized imaging proposal evaluating implanted left atrial appendage (LAA) occlusion devices by cardiac computed tomography angiography (cCTA) has never been investigated.

**Methods:**

cCTA datasets were acquired on a 3^rd^ generation dual-source CT system and reconstructed with a slice thickness of 0.5 mm. An interdisciplinary evaluation was performed by two interventional cardiologists and one radiologist on a 3D multi-planar workstation. A standardized multi-planar reconstruction algorithm was developed in order to assess relevant clinical aspects of implanted LAA occlusion devices being outlined within a pictorial essay.

**Results:**

The following clinical aspects of implanted LAA occlusion devices were evaluated within the most appropriate cCTA multi-planar reconstruction: (1) topography to neighboring structures, (2) peri-device leaks, (3) coverage of LAA lobes, (4) indirect signs of neo-endothelialization. These are illustrated within concise CT imaging examples emphasizing the potential value of the proposed cCTA imaging algorithm: Starting from anatomical cCTA planes and stepwise angulation planes perpendicular to the base of the LAA devices generates an optimal **L**AA **O**ccluder **V**iew for post-implantation **E**valuation (LOVE). Aligned true axial, sagittal and coronal LOVE planes offer a standardized and detailed evaluation of LAA occlusion devices after percutaneous implantation.

**Conclusions:**

This pictorial essay presents a standardized imaging proposal by cCTA using multi-planar reconstructions that enables systematical follow-up and comparison of patients after LAA occlusion device implantation.

**Electronic supplementary material:**

The online version of this article (doi:10.1186/s12880-016-0127-y) contains supplementary material, which is available to authorized users.

## Background

The left atrial appendage (LAA) represents the main origin of thrombus formation in atrial fibrillation, where the principles of Virchow’s triad, such as dysfunction and structural changes of the endothelium as well as abnormal blood stasis and homoeostasis are present [1–3]. The implantation of LAA occlusion devices in patients with atrial fibrillation was shown to prevent cardio-embolic stroke as safe and effective as the treatment with the oral anti-coagulant warfarin in patients eligible for oral anticoagulation [2–10]. However, the anatomic morphology of the LAA is highly variable and influences independently the incidence of cardio-embolic stroke [11, 12]. The common classification distinguishes four different LAA morphologies, which can be evaluated at best by computed tomography imaging [12–14]: 1. chicken wing, 2. cactus, 3. windsock, 4. cauliflower. All these morphologies differ extremely by the number of different LAA lobes, folds, tortuosities, diameters and global sizes and complicate individually the percutaneous implantation of LAA occlusion devices. Interestingly, the chicken wing was shown to be associated with lowest rates of cardio-embolic stroke compared to all other LAA morphologies [12].

The WATCHMAN (WM) (Boston Scientific, Natick, MA, USA) and Amplatzer Cardiac Plug (ACP) (St. Jude Medical, St Paul, MN, USA) represent the most commonly implanted LAA occlusion devices with valuable scientific evidence being available at present [2, 7, 15, 16]. Until now, trans-esophageal echocardiography (TEE) is most often applied to guide LAA occlusion device implantation, positioning and sealing both during percutaneous intervention and follow-up after device implantation [17–19]. It was shown that healing response after implantation of these devices differs and might result in harmful impact on neighboring structures [20]. However, a standardized imaging proposal for post-implantation evaluation of LAA occlusion devices by cardiac computed tomography (cCTA) has never been developed.

Therefore, this pictorial essay aims to develop this standardized imaging proposal by cCTA in order to analyze relevant clinical aspects of LAA occlusion devices after percutaneous implantation. This cCTA imaging proposal outlines relevant clinical aspects such as device positioning in relation to the varying morphology and topography of the LAA as well as functional device aspects such as peri-device leaks, lobe coverage and neo-endothelialization of the device and demonstrates illustrations of these conditions.

## Methods

### Ethics, consent and permissions

All participants of the presented patient-related data within this pictorial assays gave written consent to this analysis. The analyses were carried out according to the principles of the declaration of Helsinki and was approved by the medical ethics commission II of the Faculty of Medicine Mannheim, University of Heidelberg, Germany.

### Cardiac CTA (cCTA) protocol and image reconstruction advice for post-implantation evaluation of LAA occlusion devices

Based on our experience, cCTA protocols for the evaluation of LAA occlusion devices do not require significant protocol adjustments, when compared to standard cCTA protocols being performed for the evaluation of coronary artery stenosis [21–24]. Optimal hydration of the patient is recommended in order to achieve best measurements. Similar to a standard cCTA acquisition contrast injection should be performed with a flow rate of at least 5 cc/s followed by a saline chaser in order to have a compact contrast bolus that is washed out mainly in the right atrium and right ventricle during the image acquisition. Depending on the patients’ heart rate all available cCTA acquisition protocols in principle qualify for the evaluation of LAA occlusion devices including traditional retrospective ECG gating, prospective ECG triggering as well as high pitch or single heart beat acquisitions [25]. However, since there is no dedicated recommendation and clinical requirement for post-implantation evaluation of LAA occlusion devices in all patients, the acquisition technique that provides the lowest radiation dose depending on the available CT system should be applied. Hereby, a slightly lower image quality of the coronary arteries can be accepted since the LAA is not prone particularly to motion artifacts such as for instance the right coronary artery. Prospective ECG triggered cCTA acquisitions or single heart beat acquisitions can be used in patients with high heart rates or arrhythmias. However, different to a standard cCTA in patients with a low and regular heart rate an end-systolic image acquisition was applied for the evaluation of LAA occlusion devices in all patients independently from the heart rate in order to acquire the image data during the maximum distension of the left atrium and LAA.

Reconstruction of cCTA raw data was performed with a slice thickness between 0.5 and 0.6 mm using a sharp convolution kernel that is also used for the reconstruction of cCTA images in patients with coronary artery stent grafts or heavily calcified plaques. If available iterative reconstruction techniques should be used to lower image noise and blooming artifacts originating from metal components of the devices [26–28]. Table 1 summarizes the proposed cCTA protocol of patients after LAA occlusion device implantation.

**Table 1:** Protocol example for imaging of LAA occlusion devices using a 3^rd^ generation dual source CT system

	Heart rate <70 bpm	Heart rate 71–85 bpm	Heart rate >85	Arrhythmia
cCTA technique	High pitch single heart beat acquisition	Prospective ECG gating (step-and-shot)	Retrospective ECG gating	Retrospective ECG gating
Tube voltage	BMI <28: 70 kVp	BMI <28: 70 kVp	BMI <28: 70 kVp	BMI <28: 70 kVp
	BMI 28.1–30: 80 kVp	BMI 28.1–30: 80 kVp	BMI 28.1–30: 80 kVp	BMI 28.1–30: 80 kVp
	BMI 30.1–33: 90 kVp	BMI 30.1–33: 90 kVp	BMI 30.1–33: 90 kVp	BMI 30.1–33: 90 kVp
	BMI >33: 100–120 kVp	BMI >33: 100–120 kVp	BMI >33: 100–120 kVp	BMI >33: 100–120 kVp
Tube current-time product	Automated tube current modulation (Care Dose 4D, Siemens)	Automated tube current modulation (Care Dose 4D, Siemens)	Automated tube current modulation (Care Dose 4D, Siemens)	Automated tube current modulation (Care Dose 4D, Siemens)
Slice thickness	0.6 mm	0.6 mm	0.6 mm	0.6 mm
Reconstruction increment	0.4	0.4	0.4	0.4
Reconstruction kernel	Bv40 (vascular kernel) (Siemens)	Bv40 (vascular kernel) (Siemens)	Bv40 (vascular kernel) (Siemens)	Bv40 (vascular kernel) (Siemens)
Reconstruction phase	70 % RR	40–70 % RR	20–70 % RR	10–90 % RR
Reconstruction technique	Iterative reconstruction level III (ADMIRE, Siemens)	Iterative reconstruction level III (ADMIRE, Siemens)	Iterative reconstruction level III (ADMIRE, Siemens)	Iterative reconstruction level III (ADMIRE, Siemens)
Contrast medium	80 cc iomeprol 400 (Bracco)^a^	80 cc iomeprol 400 (Bracco)^a^	80 cc iomeprol 400 (Bracco)^a^	80 cc iomeprol 400 (Bracco)^a^

Note: bpm: beats per minute; kVp: kilovoltage peak; ECG: electrocardiogram^a^ contrast material is not reduced as it is possible for a standard coronary CT angiography in order to reduce blood stasis artifacts within the left atrial appendage; the scan start is determined using bolus tracking within the descending aorta using a threshold of 120 HU and an additional delay of 7 s in order to have a slightly delayed scan start when compared to standard

### Generating a standardized proposal by multi-planar imaging

**Fig. 1:**
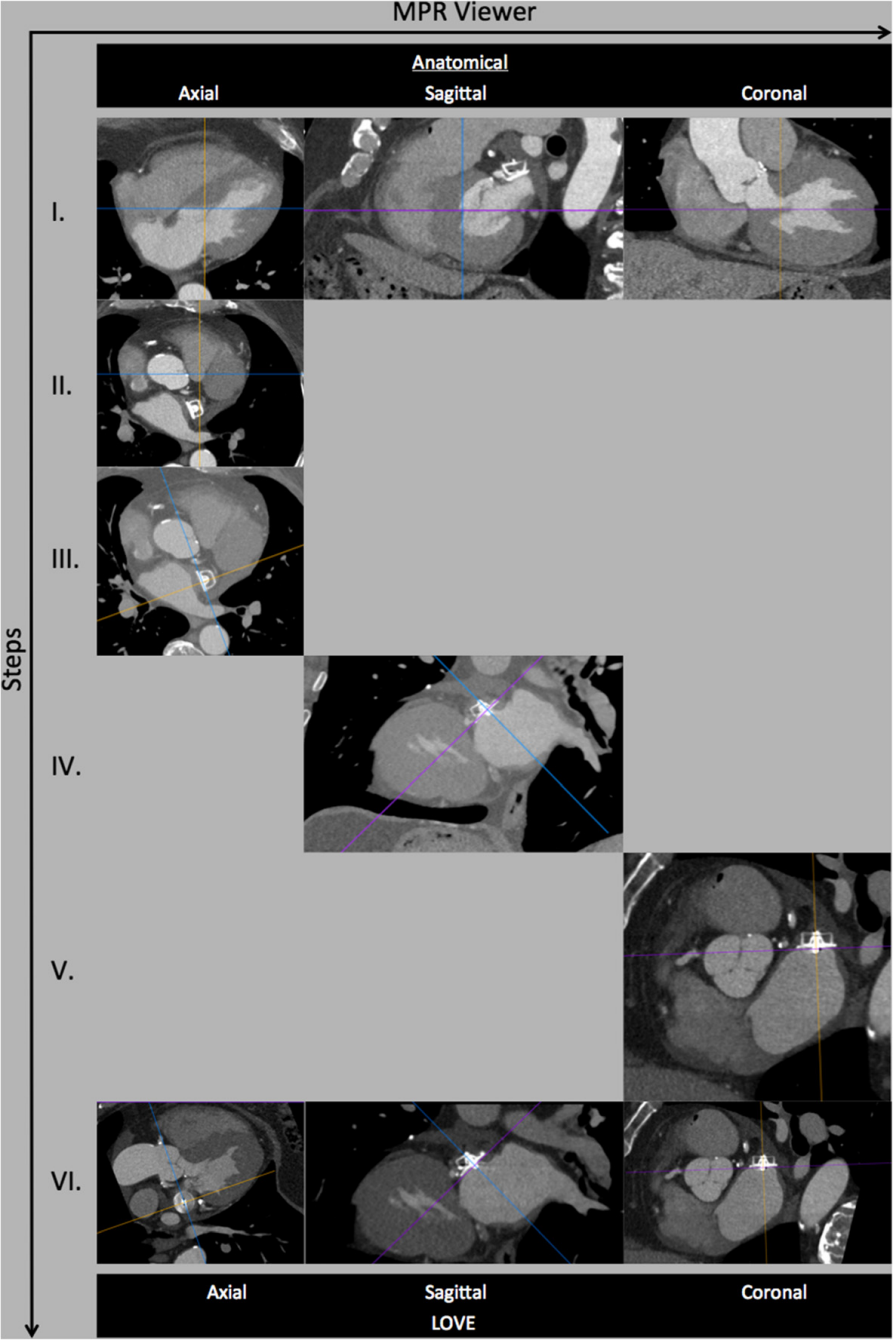
Illustration of a stepwise standard multimodal imaging to generating optimal LAA Occluder View for post-implantation Evaluation (LOVE) planes from anatomical cCTA planes. Figure shows 5 standardized steps (panels I-V) for the ACP® device

**Fig. 2:**
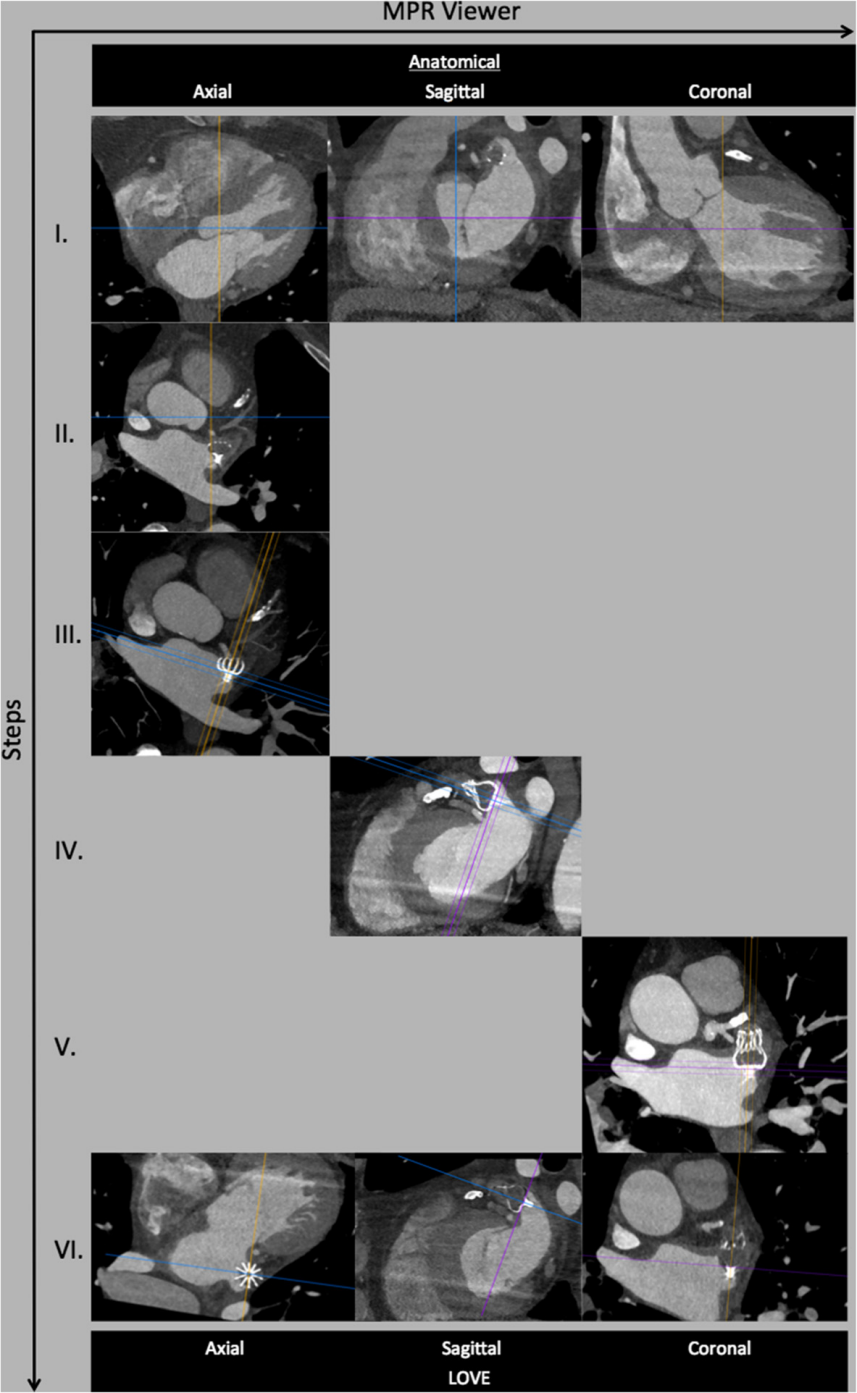
Illustration of a stepwise standard multimodal imaging to generating optimal LAA Occluder View for post-implantation Evaluation (LOVE) planes from anatomical cCTA planes. Figure shows 5 standardized steps (panels I-V) for the WATCHMAN® device

0.6 mm thick images reconstructed with a medium convolution kernel are uploaded on a simple 3D multi-planar rendering workstation with the 3 planes locked at a 90° angle (Figs. 1 and 2, panels I) (Additional files 1 and 2). The axial slices should be moved to the level of the left atrium, in which the LAA occlusion device is visible (Figs. 1 and 2, panels II). These initial steps are independent of different type of implanted LAA occlusion devices (either WM or ACP).

### Amplatzer cardiac plug (ACP) device

Specifically for the ACP device, move the coronal axis within the transverse window perpendicular to the disc of the ACP device (Fig. 1, panel III). Afterwards, align the axes on the two other viewers also perpendicular to the disc of the ACP device (Fig. 1, panels IV and V). Lastly, the center of the axes should be placed to the center of the screw-hub.

### WATCHMAN (WM) device

Since the WM device is not equipped with a disc, the alignment might become more challenging. Here, using maximum intensity projection (MIP) images with a slab thickness of approximately 10 mm allows visualization of the whole nitinol frame of the WM device. Afterwards, move the coronal axis within the transverse window perpendicular to the coves of the parachute of the WM device (Fig. 1, panel III). Afterwards, align the axes on the two other viewers also perpendicular to the coves of the parachute of the ACP device (Fig. 1, panels IV and V). Lastly, the center of the axes should be placed to the center of the screw-hub.

Starting from anatomical cCTA planes while applying all of the described imaging steps above will generate the optimal **L**AA **O**ccluder **V**iew for post-implantation **E**valuation (LOVE) (Figs. 1 and 2, Additional files 1, 2, 3 and 4). This standardized imaging reconstruction view can be applied for the two most commonly implanted LAA occlusion devices (i.e. ACP and WM). The LOVE view allows optimal evaluation of the most relevant clinical aspects of the post-implantation follow-up of patients with LAA occlusion devices: (1) peri-device leaks, (2) coverage of LAA lobes, (3) indirect signs of neo-endothelialization.

## Results

### Clinical scenarios reflecting relevant clinical aspects after implantation of LAA occlusion devices

Imaging reports about percutaneously implanted LAA occlusion devices should focus on the following important clinical aspects:

### Morphologic and topographic aspects of LAA occlusion device positioning

**Fig. 3:**
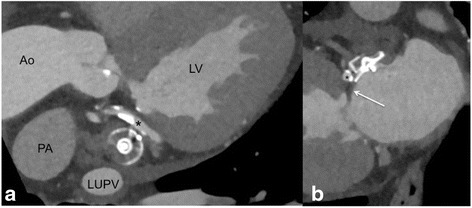
LOVE coronal (panel **a**) and sagittal (panel **b**) reformations demonstrating the anatomic relationship to relevant neighboring structures that should be reported. Panel **a** shows the close anatomic relationship to the left upper pulmonary vein (LUPV) and the left circumflex coronary artery (LCX) that is adjacent directly to the LAA occlusion device (* in panels **a** and **b**). The pulmonary artery (PA) is the third relatively close neighboring structure that should be inspected carefully on LOVE axial reformations

- Global positioning of the device including rotation around the entry axis to the LAA. This can be best measured on LOVE axial and LOVE sagittal views.
- LOVE axial views allow optimal evaluation of global compression of both devices:
  WM device: compression of 10–20 % referred to the original device size is recommended.
  ACP device: concave disc; device lobe positioned 2/3 distal to the LCX inside and engaged within the LAA; ACP device axis in line with the LAA neck axis.
- LOVE axial views allow optimal evaluation of impairment to neighboring structures by the LAA occlusion device: including positioning in relation to the mitral valve annulus, pulmonary artery, left pulmonary veins as well as LCX that is best visible in LOVE axial views (Fig. 3).

### Functional aspects of LAA occlusion devices

**Fig. 4:**
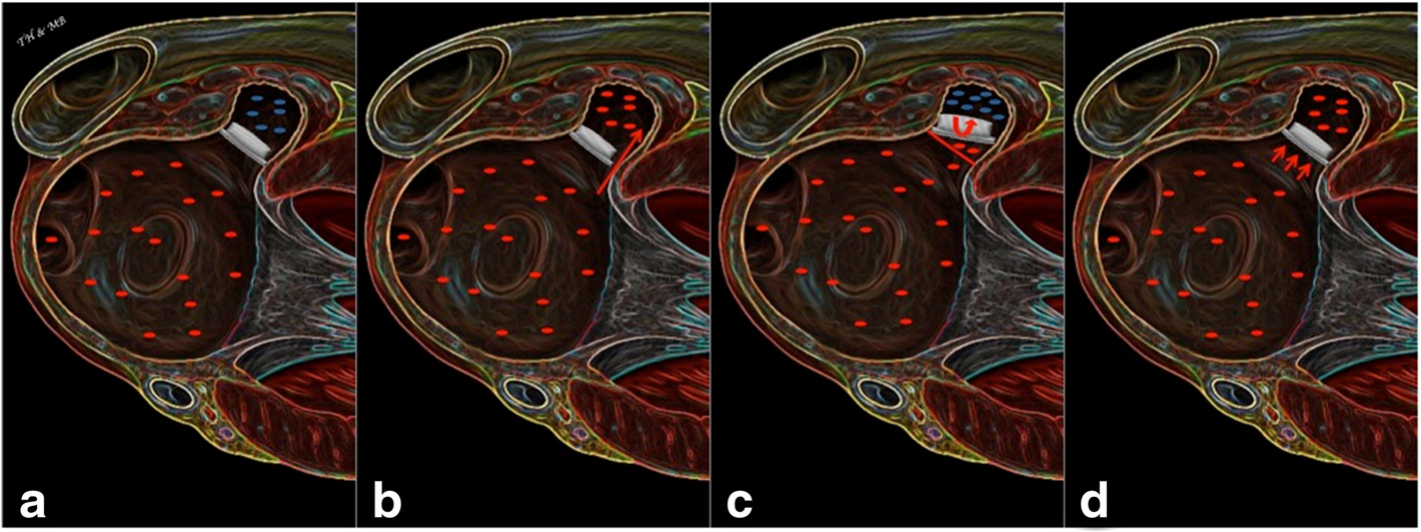
The figure summarizes schematically the four main follow-up scenarios on cardiac CTA after LAA occlusion device implantation. Panel **a** demonstrates optimal positioning of the LAA occlusion device without any residual lobe, any peri-device leak and without any residual contrast filling (blue dots in LAA) indicating complete neo-endothelialization. Panel **b** demonstrates a peri-device leak with contrast filling of the LAA. Panel **c** shows sub-optimal positioning of the LAA occlusion device with in-complete lobe coverage leaving a residual left atrial appendage. Panel **d** shows optimal positioning of the LAA occlusion device with in-complete neo-endothelialization that is suggested indirectly by the contrast enhancement of the LAA (blue dots in LAA). Please note that all three scenarios can occur in combination. In case of an existing peri-device leak with contrast filling of the LAA assessment of endothelialization is not feasible with cardiac CT

**Fig. 5:**
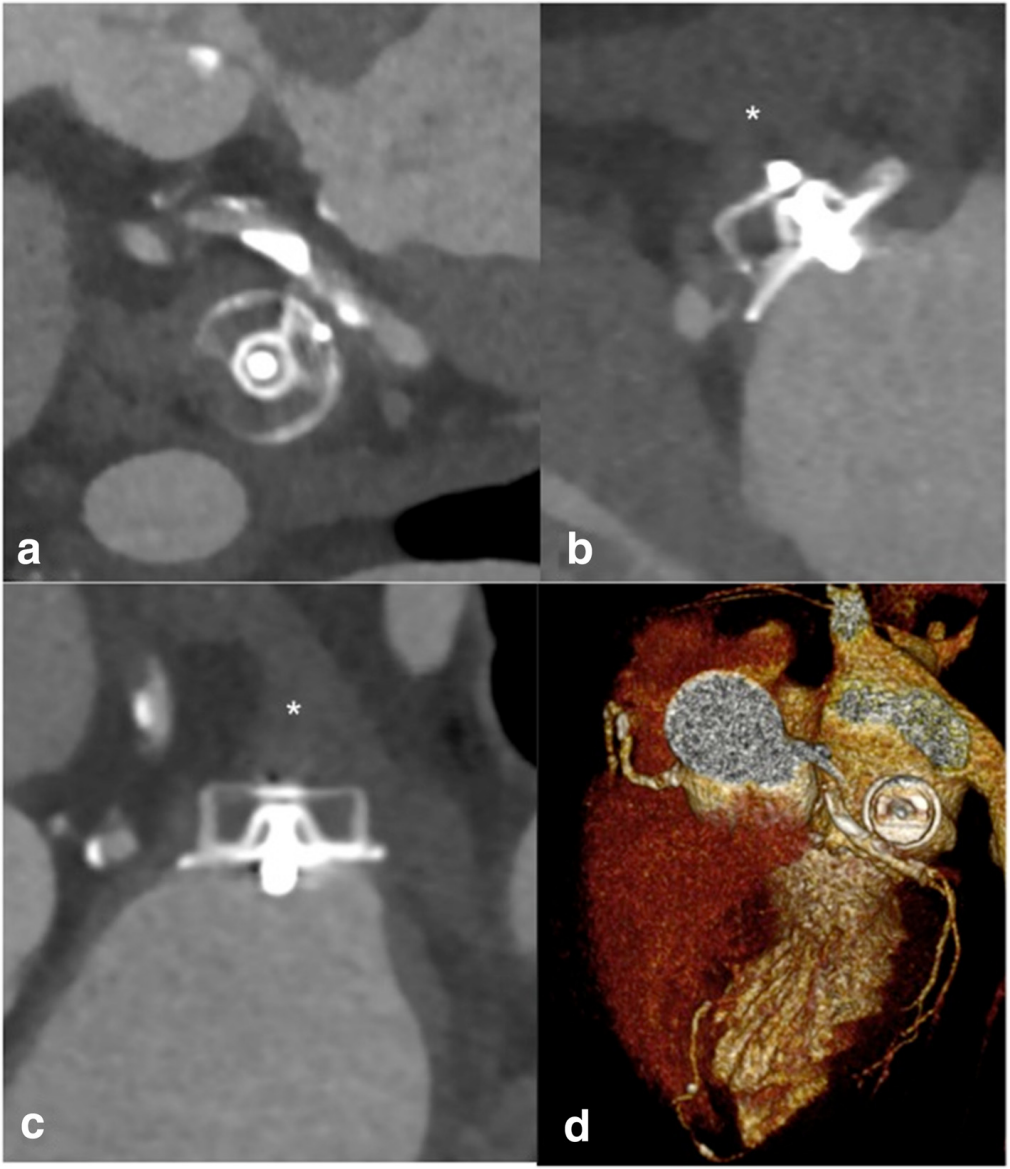
LOVE - axial (panel **a**), − sagittal (panel **b**), - coronal (panel **c**), and 3D (panel **d**) reformations of a 72 year-old male patient who underwent cardiac CTA 9 months after implantation of a ACP® LAA occlusion device. Panels **a**, **b**, and **c** show optimal positioning of the device with no peri-device leak, complete lobe coverage as well as complete neo-endothelialization of the device suggested by the absence of contrast enhancement < 50 Hounsfield units in the LAA (* in B and C). Volume rendered reconstructions

**Fig. 6:**
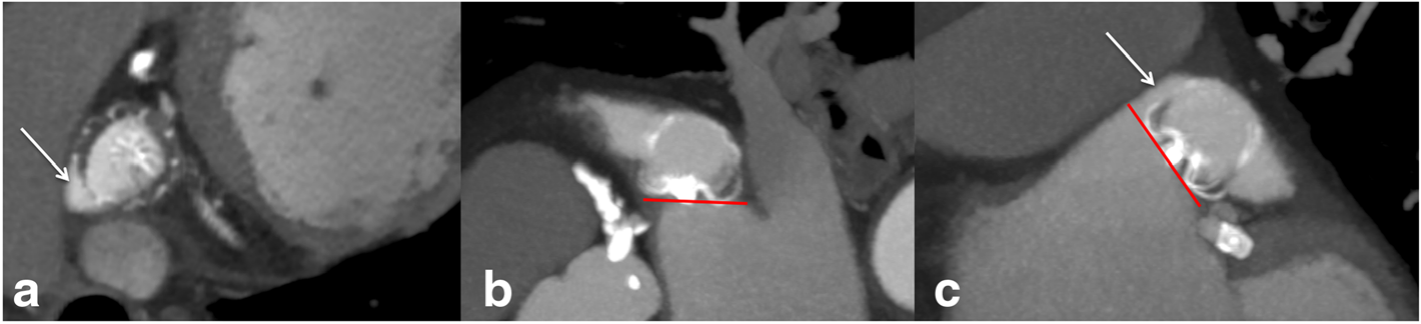
LOVE - axial (panel **a**), − sagittal (panel **b**), and - coronal (panel **c**) reformations of a 80-year-old male patient who underwent cardiac CTA 8 months after implantation of a WATCHMAN® LAA occlusion device. Panels **a** and **c** show a small peri-device leak <3 mm at the cranial LAA entry causing complete contrast filling of the LAA (*white arrow*). Panel **b** demonstrates the correct positioning of the device with complete lobe coverage, as indicated by the correctly positioned sealing part (proximal cap of the WM device) parallel to the plane of the LAA ostium (*red line*). Due to the presence of the peri-device leak the assessment of endothelialization is not feasible with cardiac CTA
- Coverage of all lobes:
  The LAA often consists of different lobes not corresponding necessarily to the fixed shape of the LAA occlusion device. Therefore, complete coverage of all LAA lobes is not always achievable. Assessment of lobe coverage should consider the angle between the sealing part of the device (either proximal disk or cap) and the plane of the LAA ostium. This angle corresponds to the incompletely covered LAA lobe. Accordingly, Fig. 7 illustrates a small residual LAA lobe due to over-angulation of the device. Rotation around the entry axis to the LAA should be measured on LOVE axial and LOVE sagittal views. Lobe coverage should be assessed on all LOVE reformations (Figs. 4, 5, 7 and 8).

- Peri-device leaks:
  These can be seen best on LOVE sagittal views. The presence of a peri-device leak should be measured in all reports (Figs. 4, 5 and 6). Higher resolution measurements of peri-device leak diameters can be performed by cCTA compared to TEE. Accordingly, further studies are needed to evaluate the additional value of cCTA measurements in this context and whether definitions of the PROTECT AF study can accordingly be transferred (i.e. minor (<1 mm width), moderate (1 to 3 mm width) or major (3 mm width)). A sole peri-device leak should be reported in case the sealing part of the device (either proximal disk or cap) is parallel to the plane of the LAA ostium. Remaining contrast filling will then be visible around the device on the LOVE views (Fig. 6).

**Fig. 7:**
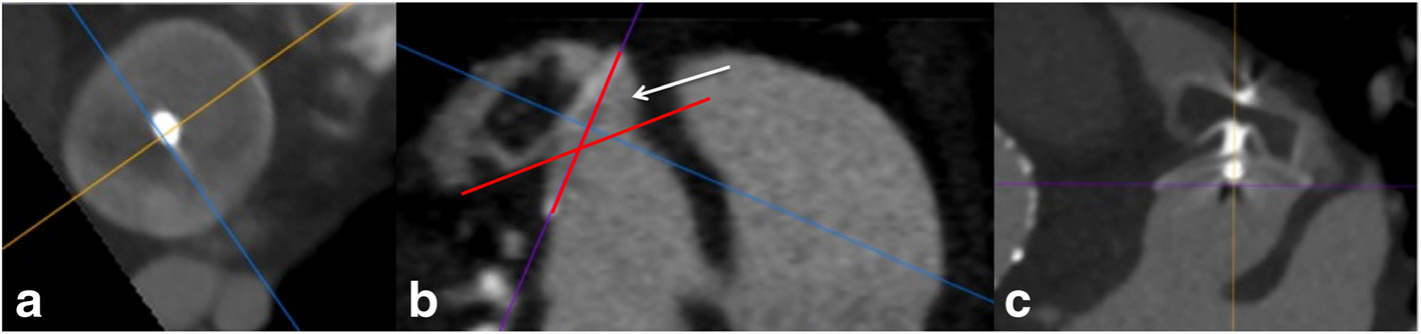
LOVE - axial (panel **a**), − sagittal (panel **b**), and - coronal (panel **c**) reformations of a 83-year-old female patient who underwent cardiac CTA 9 months after implantation of the ACP® LAA occlusion device. Panels **a**, **b** and, **c** demonstrate no peri-device leak. Panel **b** shows incomplete lobe coverage with a small residual LAA lobe. The uncovered LAA lobe is seen between the angle of the sealing part of the ACP device (proximal disk) and the plane of the LAA ostium (angle in between *red lines*, marked by *white arrow*). The contrast filling of the LAA in combination with the clear absence of a peri-device leak reflects indirectly in-complete endothelialization of the ACP® LAA occlusion device

**Fig. 8:**
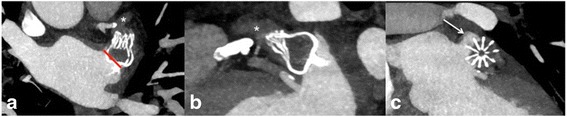
LOVE - axial (panel **a**), − sagittal (panel **b**), and - coronal (panel **c**) reformations of a 75-year-old male patient who underwent cardiac CTA 4 months after implantation of a WATCHMAN® LAA occlusion device. Panel **a** shows a 20° offset of the device around the entry axis to the LAA (red line in A). The rotation led to an in-complete lobe coverage with a small residual left atrial appendage < 5 mm that is best seen on the LOVE coronal reformation (arrow in panel **c**). The residual slight contrast enhancement (* in panels a and b) of the LAA that is approximately 50 % lower when compared to the contrast enhancement in the left atrium suggests beginning, but still in-complete neo-endothelialization of the device
- Complete neo-endothelialization:
  Absence of contrast enhancement within the LAA without any peri-device leak suggests complete neo-endothelialization. Accordingly, contrast enhancement in the LAA of less than 50 Hounsfield units compared to the left atrium suggests incomplete neo-endothelialization. Equal contrast enhancement in both LAA and left atrium suggests no or very early stages of neo-endothelialization (Figs. 4, 5, 7 and 8).

## Discussion

This pictorial essay presents a standardized imaging proposal by cCTA using multi-planar reconstructions that enables systematical follow-up and comparison of patients after LAA occlusion device implantation. As described above and being accompanied by a case series of striking illustrations this imaging proposal intends to cover the most relevant clinical challenges of implanted LAA occlusion devices including morphologic, topographic as well as functional device-related aspects. The presented imaging proposal generates novel hypotheses, which have to be proven by ongoing prospective, randomized imaging studies.

Based on the so far available literature on safety and patient outcome, it is likely that the number of patients undergoing percutaneous LAA occlusion device implantation will raise significantly within the near future similar to the growing number of patients undergoing transcatheter aortic valve replacement (TAVR). However, in order to identify patients with poor outcome and/or post-procedural complications, such as a residual LAA larger than a specific device size that still leads to thrombus formation, accurate, reproducible and reader-independent imaging is crucial. This is of particular importance in order to generate more evidence about optimal placement of LAA occlusion devices.

For instance, the presence of relevant peri-device leaks or incomplete coverage of residual LAA lobes might be associated with individually impaired neurologic outcome of the patients. These mal-appositions of LAA occlusion devices have been attributed to clinical overt neurologic disability and silent cerebral ischemia being caused by cerebral micro-embolism and visualized by cerebral magnetic resonance imaging (cMRI) [17]. Importantly, cCTA might also allow the evaluation of complete neo-endothelialization of the LAA occlusion device. From the treating physician’s perspective complete neo-endothelialization without any peri-device leak might facilitate future clinical decision-making to stop concomitant anticoagulation or dual antiplatelet therapy (DAPT).

Little is known regarding incidences of device related complications after successful percutaneous implantation of LAA occlusion devices, such as device compression, peri-device leaks, lobe coverage and progress of neo-endothelialization related to study-specific antithrombotic treatments. The latter were evaluated for DAPT (i.e. aspirin and clopidogrel) lasting 6 months, as within the present study, or for warfarin for 45 days, followed by clopidogrel for 4.5 months and lifelong aspirin [2, 7, 10, 16]. There is a lack of data comparing the quality of TEE versus CT follow-up imaging after device implantation and the optimal time period still needs to be investigated. Device compression and shape in relation to surrounding vessels is of importance for optimal device and hemodynamic stability over time in order to prevent relevant dislodgment or obstruction of neighboring structures as being recommended by the manufacturer [29]. It was shown recently, that compression and shape of LAA occlusion devices are changing temporarily within three months after implantation, however its clinical relevance besides complete LAA closure is still under debate [30, 31].

Furthermore, accurate differentiation between thrombus formation and blood stasis within the LAA is challenging using standard cCTA. An additional delayed cCTA approximately 70 s after the start of the contrast injection is recommended [32]. Imaging the patients in prone position is another theoretical approach in order to minimize the effects of blood stasis in the LAA. However, data is lacking about the usefulness of performing cCTA in a prone position. Dual energy cCTA using calculated iodine maps has been proposed in order to differentiate accurately blood stasis from thrombus formation within the LAA of patients with cardio-embolic stroke [33].

As outlined, post procedural image analysis with cCTA might bear the potential of a standardized imaging using defined multi-planar reformations that best display the complex 3-dimensional shape of the LAA and the device. In our opinion, cCTA has the main advantage that the images can be very standardized analyzed and reconstructed and make quantitative measurements reliable. This is of particular importance to generate more evidence on relevant clinical questions: (1) What size of a residual LAA is acceptable with different devices? (2) What size of peri-device leaks still leads to a complete occlusion over time? However, the advantages of cCTA for post-implantation LAA occlusion device evaluation have to outscore the patients’ individual risk being associated with an additional radiation dose and additional administration of iodinated material.

### Potential value and limitations of cardiac CTA in the context of LAA occlusion devices

Cardiac CTA allows the comprehensive non-invasive visualization of the whole heart including coronary arteries as well as all important neighboring structures of the LAA and thus accurate evaluation of post-implantation evaluation of LAA occlusion devices by the described LOVE view. The main arguments for the use of cCTA in this context, besides its non-invasiveness, are firstly, the whole volume coverage with a high spatial resolution and secondly, the high reproducibility of the technique that allows to angulate the isotropic dataset retrospectively after the procedure in all desired projections. Thereby generating an investigator’s independency, cCTA becomes particularly attractive for clinical studies that require a high standardization of follow-up examinations. Thirdly, image artifacts do not hamper cCTA image quality because all available LAA occlusion devices do not lead to metal artifacts such as other metal implants. Based on our experience, cCTA allows assessing all relevant clinical questions including indirect visualization of LAA occlusion device neo-endothelialization if no peri-device leak is present. For instance, no contrast enhancement within the LAA suggests complete neo-endothelialization, whereas contrast enhancement in the LAA of less than 50 Hounsfield units compared to the enhancement within the left atrium suggests incomplete neo-endothelialization. Contrast enhancement within the LAA that is equal to the left atrium suggests no or very early stages of neo-endothelialization.

The main concerns with cCTA in the context of LAA occlusion device evaluation are the additional radiation dose that is given to the patient. However, with low radiation dose techniques of state-of-the-art CT systems the radiation dose has been reduced significantly over the past 5 years [25]. Thus, the risk of ionizing radiation in this mainly elderly population should be weighted clinically against potential overseen significant clinical findings potentially being detected by cCTA (such as thrombus formation or indirect signs of incomplete neo-endothelialization), whereas their clinical impact still needs to be proven within prospective clinical studies.

The second relevant concern of cCTA is the need for iodinated contrast material, which is crucial in patients with impairment of renal function. Although the amount of contrast material for the evaluation of LAA occlusion devices can be reduced to a minimum of 30–40 cc of contrast in patients with elevated serum creatinine levels, the indication should be checked strictly in patients with serum creatinine levels above 1.5 mg/dL. Additionally, cCTA is more expensive and not as widely available compared to echocardiography.

## Conclusions

This pictorial essay describes a standardized imaging proposal including examples of (ab)normal findings after percutaneous implantation of LAA occlusion devices. Whether the proposed cCTA imaging instructions might facilitate future clinical follow-up, cost effectiveness and therapeutic decisions needs to be evaluated further in larger, prospective and randomized imaging studies evaluating this specific subset of cardiac patients.

## Additional files

Additional file 1: Movie demonstrating the generation of the **L**AA **O**ccluder **V**iew for post-implantation Evaluation (LOVE) for the Amplatzer cardiac plug (ACP) device by cardiac CTA. (MP4 7602 kb) Additional file 2: Movie demonstrating the generation of the **L**AA **O**ccluder **V**iew for post-implantation Evaluation (LOVE) for the WATCHMAN (WM) device by cardiac CTA. (MP4 8524 kb) Additional file 3: Movie revealing the potential diagnostic insights of cardiac CTA imaging for an implanted ACP device by optimal alignment of LOVE planes. (MP4 4653 kb) Additional file 4: 3D animation illustrating optimal implantation of an ACP device within the LAA after optimal alignment of LOVE planes by cardiac CTA. (MP4 3466 kb)

## Abbreviations

ACP: amplatz cardiac plug
BMBF: German Ministry of Education and Research
cCTA: cardiac computed tomography
cMRT: cerebral magnetic resonance imaging
CT: computed tomography
DAPT: dual antiplatelet therapy
DZHK: Deutsches Zentrum für Herz-Kreislauf-Forschung - German Centre for Cardiovascular Research
LAA: left atrial appendage
LOVE: LAA occluder view for post-implantation evaluation
MIP: maximum intensity projection
TEE: transesophageal echocardiography
WM: Watchman
